# Diffusion model for imputing time-series gut microbiome profiles using phylogenetic information and metadata integration

**DOI:** 10.1093/bioadv/vbaf181

**Published:** 2025-07-28

**Authors:** Misato Seki, Yao-zhong Zhang, Seiya Imoto

**Affiliations:** Division of Health Medical Intelligence, Human Genome Center, The Institute of Medical Science, The University of Tokyo, Tokyo 108-8639, Japan; Division of Health Medical Intelligence, Human Genome Center, The Institute of Medical Science, The University of Tokyo, Tokyo 108-8639, Japan; Division of Health Medical Intelligence, Human Genome Center, The Institute of Medical Science, The University of Tokyo, Tokyo 108-8639, Japan

## Abstract

**Motivation:**

The gut microbiota interacts closely with the host, playing crucial roles in maintaining health. Analysing time-series genomic data enables the investigation of dynamic microbiota changes. However, missing values create significant analytical challenges.

**Results:**

We propose a microbiome imputation framework based on a conditional score-based diffusion model, tailored to microbiome data by incorporating phylogenetic convolutional layers. Our method effectively reduces mean absolute error across various missing data ratios for both 16S rRNA and whole-genome shotgun profiles. The imputed datasets enhance downstream predictive tasks, achieving area under the curve scores that exceed or are comparable with those of the existing methods. To further improve the performance, we embedded host metadata into the model using a tabular encoding approach, which yielded additional improvements particularly under higher missing ratios. Our findings underscore the potential of the diffusion model for processing time-series microbiome data with missing values.

**Availability and implementation:**

Related codes and dataset can be found at: https://github.com/misatoseki/metag_time_impute_phylo.git.

## 1 Introduction

The human gut harbors a diverse and highly complex community of microbiota that plays a crucial role in maintaining host health. These microorganisms influence various physiological processes, including modulating immune responses (Peroni *et al.* 2020), supporting metabolic functions (Tilg *et al.* 2011), and impacting nervous system activity, including mental health (Dinan and Cryan 2017). Gaining a deeper understanding of these interactions offers considerable potential for advancing therapeutic strategies to treat and prevent diseases across a wide range of medical fields.

Recent advancements in genome sequencing technology have substantially accelerated gut microbiota research. These technologies enable the investigation of uncultivable bacteria thriving in the gut’s anaerobic environment. While 16S ribosomal RNA (rRNA) sequencing provides insights into the composition of microbial communities, whole-genome shotgun (WGS) sequencing extends this by offering both compositional data and functional gene analysis. These rich datasets are actively analysed using deep learning technologies, which can rapidly process large datasets. For instance, for predicting diseases based on gut microbiota, deep representation learning using autoencoders (Oh and Zhang 2020), deep learning combining known bacteria features and unknown sequences (Chen *et al.* 2022), self-knowledge distillation-driven convolutional neural network (CNN)-long short-term memory (LSTM) models (Fung *et al.* 2023) have recently been developed.

Analysing time-series microbiome data is crucial for elucidating temporal variations in microbial composition and their interactions with host factors such as aging, diet, medication, and illness (David *et al.* 2014, Weersma *et al.* 2020, Bosco and Noti 2021). However, a major challenge in such analyses is handling missing time points (Kodikara *et al.* 2022). Capturing longitudinal changes accurately is essential for identifying microbial patterns associated with health or disease progression, and several methods have been developed to address this issue. In the deep learning model for disease prediction from time-series metagenomic data (Sharma and Xu 2021), the missing time points were handled with masking techniques. Additionally, DeepMicroGen, an imputation method based on LSTM and generative adversarial networks (GAN) (Choi *et al.* 2023) was introduced to predict the composition at missing time points. While DeepMicroGen improves performance by imputing missing values, in the context of GANs, challenges such as training instability have been reported (Pan *et al.* 2019).

**Figure 1.**
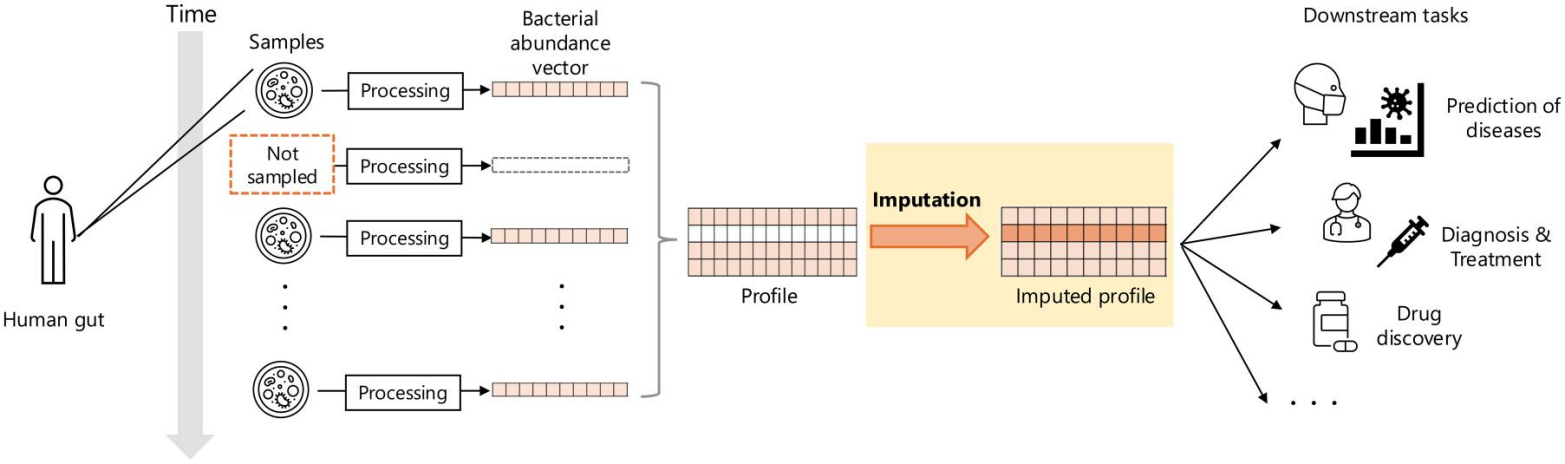
Outline of the study. Time-series human gut samples often include missing time points, presenting challenges for downstream analyses. The proposed method aims to impute missing bacterial abundance profiles based on observed time points, thereby providing complete metagenomic profiles. This approach is expected to enhance the accuracy and utility of downstream tasks involving these datasets.

The diffusion model, a state-of-the-art generative model, has demonstrated stable and superior performance compared with other models, including GAN. It has been increasingly applied to tasks such as image synthesis, natural language processing, and time-series prediction and imputation (Alcaraz and Strodthoff 2022, Yang *et al.* 2023, Lin *et al.* 2024). In this study, we propose a diffusion model–based imputation method to reconstruct microbiota abundance profiles. Specifically, our approach uses the Conditional score-based diffusion models for probabilistic time series imputation (CSDI) (Tashiro *et al.* 2021), which has shown notable performance on 16S rRNA sequencing data (Seki *et al.* 2023). Our method incorporates a modified denoising function leveraging phylogenetic categories to adapt it to microbiome data. We evaluate its performance on multiple datasets, including 16S rRNA and WGS sequencing, and demonstrate its utility in predicting disease presence using imputed profiles as a downstream biological application. The workflow is outlined in Fig. 1. Furthermore, inspired by CSDI_T (Zheng and Charoenphakdee 2022), a variant of CSDI designed for tabular data, we enhanced the model by integrating metadata information using a feature tokenizer (FT) (Gorishniy *et al.* 2021), aiming to further improve imputation performance. This research aims to facilitate the broader use of incomplete time-series microbial profiles, enhancing the understanding of microbial communities and their implications for human health.

## 2 Methods

### 2.1 Conditional diffusion model for probabilistic time-series imputation

The diffusion model is a family of probabilistic generative models that generates outputs by progressively denoising random noise (Yang *et al.* 2023). The model consists of two iteration processes: the *Forward process*, which progressively destructs original data by adding noise until it becomes completely unstructured, and the *Reverse process*, which progressively reconstructs the original data by removing noise. Given that $p_{\theta }(x_{0})$ approximates the data distribution $q(x_{0})$ for the data $x_{0}$ and that $x_{t}$ is a sequence of latent variables for iteration process of t=1,…,T, the reverse process can be described as the joint distribution, that is modeled as a Markov chain with Gaussian transitions starting at $p(x_{T})=N(x_{T};0,I)$:


(1) $$p_{\theta }(x_{0:T}):=p(x_{T})\Pi_{t=1}^{T}p_{\theta }(x_{t-1}|x_{t}),$$

(2) $$p_{\theta }(x_{t-1}|x_{t}):=N(x_{t-1};\mu_{\theta }(x_{t},t),\sigma_{\theta }(x_{t},t)I).$$

The parameterization of $p_{\theta }(x_{t-1}|x_{t})$ follows the denoising diffusion probabilistic models framework (Ho *et al.* 2020).

**Figure 2.**
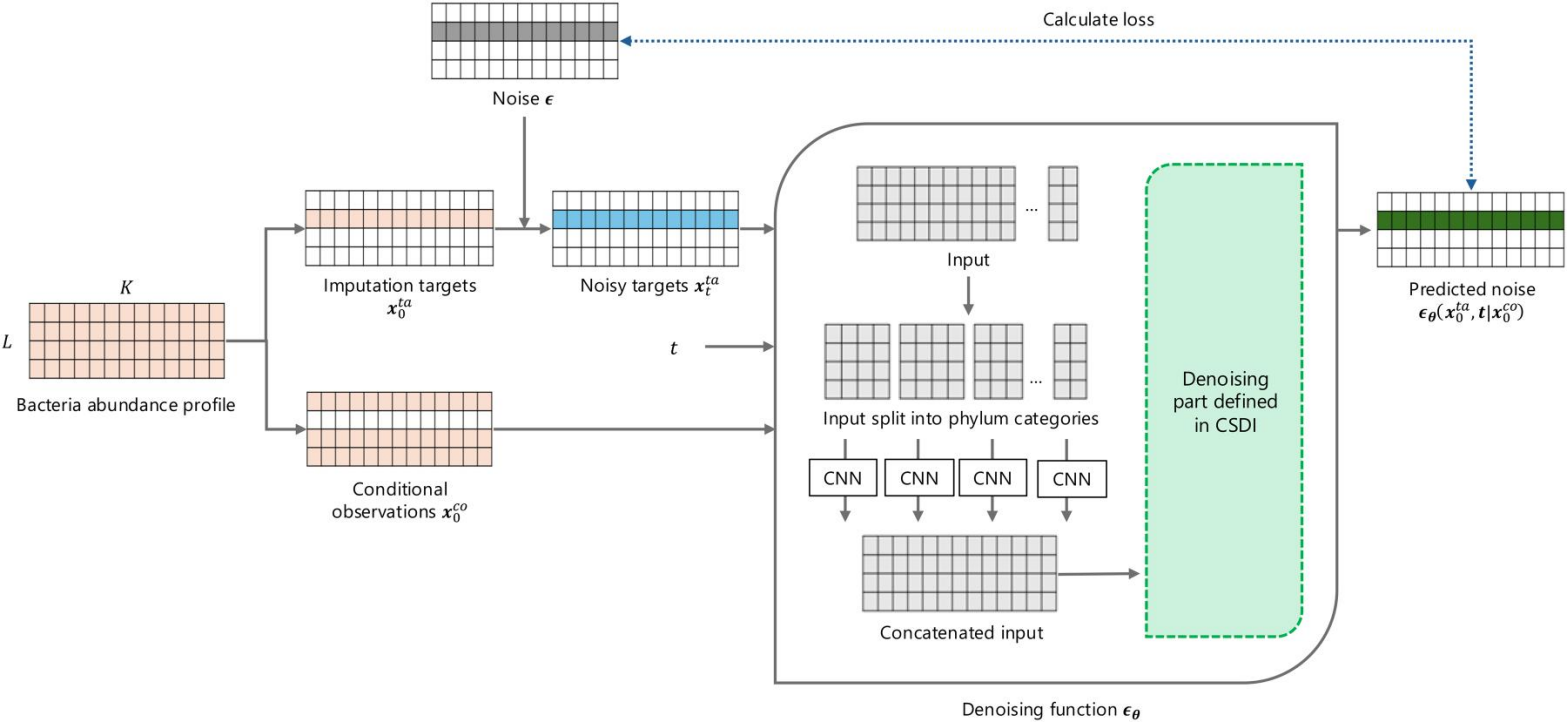
Outline of the model structure and self-supervised training process of the proposed method based on the CSDI framework (Tashiro *et al.* 2021). Phylogenetic information is incorporated into the denoising function via CNNs for each phylum category.

To implement this diffusion model in our imputation approach, we utilize the CSDI framework, which employs a diffusion model conditioned on observed data (Tashiro *et al.* 2021). In this model, to conduct the self-supervised learning, the data is split into the conditional observations $x_{0}^{co}$ and the imputation targets $x_{0}^{ta}$ with specific ratios. An overview of the self-supervised training is illustrated in Fig. 2. The conditional reverse process can be described as following:


(3) $$p_{\theta }(x_{0:T}^{ta}|x_{0}^{co}):=p(x_{T}^{ta})\Pi_{t=1}^{T}p_{\theta }(x_{t-1}^{ta}|x_{t}^{ta},x_{0}^{co}),$$

(4) $$p_{\theta }(x_{t-1}^{ta}|x_{t}^{ta},x_{0}^{co}):=N(x_{t-1}^{ta};\mu_{\theta }(x_{t}^{ta},t|x_{0}^{co}),\sigma_{\theta }(x_{t}^{ta},t|x_{0}^{co})I).$$

The parameters θ are learned by maximizing the evidence lower bound. This can be achieved by minimizing the loss function through the following process, which is defined to fit the conditional model:


(5) $$\underset{\theta }{\min}L(\theta )=\underset{\theta }{\min}E_{x_{0},\epsilon ,t}||(\epsilon -\epsilon_{\theta }(x_{t}^{ta},t|x_{0}^{co}))|{|}_{2}^{2},$$

where $\epsilon_{\theta }$ is a trainable denoising function and ϵ is a noise vector following a standard normal distribution. Additionally, one-layer transformer encoders are included for each of temporal and feature dimension to take account of dependencies within each dimension (Tashiro *et al.* 2021).

### 2.2 Implementation on bacteria abundance datasets

The bacteria abundance profile for the *i*th subject can be represented as $x_{0}^{\left(i\right)}=\{x_{l,k}\}$, where l∈{1,…,L} denotes time points and k∈{1,…,K} denotes bacteria species. The bacteria species were ordered based on their correlation to place correlated features together, following the method proposed in the previous studies (Sharma *et al.* 2020, Sharma and Xu 2021). The operational taxonomic units (OTUs) were split into phylum categories and Spearman’s correlation coefficient ρ was calculated between all pairs of OTUs within the category. Geometric mean $\rho_{OTU_{j}}$ was calculated for *j*th OTU as following:


(6) $$\rho_{{\text{OTU}}_{j}}=\sqrt[{p}]{\left|\rho_{{\text{OTU}}_{j1}}\cdot \rho_{{\text{OTU}}_{j2}}\cdot \ldots \cdot \rho_{{\text{OTU}}_{jp}}\right|},1\leq j\leq p,$$

where $\rho_{OTU_{jp}}$ was Spearman’s correlation between OTU_*j*_ and OTU_*p*_. The geometric means were sorted in descending order within the category. To prevent information leakage, ordering was performed using only the training set, ensuring that the test set remained unseen. To incorporate phylogenetic information, convolutional layers were added for each phylum category in the denoising functions, building on previous research (Choi *et al.* 2023). Feature vectors generated for each phylum category were concatenated. The structure of the CNN layers in the denoising function is illustrated in Fig. 2. A rectifier activation function follows the CNN layer.

### 2.3 Dataset

We used two time-series bacterial abundance datasets for our experiments, with general information summarized in Supplementary Table 1, available as supplementary data at *Bioinformatics Advances* online. For all dataset used in this study, relative abundance values below 0.01% were replaced with 0% to exclude false positives, and the values were transformed using the centered log ratio (clr) prior to model training (Gloor *et al.* 2017). To handle zero values before clr transformation, a pseudo-count equal to half of the minimum abundance value was added.

#### 2.3.1 16S rRNA data from DIABIMMUNE study

We employed the 16S rRNA profile of gut microbiota from the three-country cohort of the DIABIMMUNE project (Vatanen *et al.* 2016), which contains fecal samples from infants in Estonia, Finland, and Russia. The relative abundance data, preprocessed using QIIME 1 (Caporaso *et al.* 2010), along with the corresponding metadata table, were obtained from the publicly accessible repository of the project (https://diabimmune.broadinstitute.org/diabimmune/). Sample collection was organized into 6-month intervals. Of the 222 subjects enrolled in the study, a total of 116 subjects were selected for our analysis, each with five samples at 0, 6, 12, 18, and 24 months of age. The resulting profiles contain 113 OTUs at the species level.

#### 2.3.2 WGS data from BONUS study

The Baby Observational and Nutritional Study (BONUS) provided time-series fecal samples from 207 infants with cystic fibrosis, obtained using WGS metagenomic sequencing (Hayden *et al.* 2020). Samples were collected at seven time points: 3, 4, 5, 6, 8, 10, and 12 months of age. We generated the relative abundance profile using MetaPhlAn 4 (Blanco-Míguez *et al.* 2023) from sequencing data obtained from Sequence Read Archive at the National Center for Biotechnology Information (NCBI) under BioProject accession PRJNA510445. Subjects with data from five or more time points were included, resulting in a subset of 157 subjects with a missing rate of 11% and 833 features at the species level.

### 2.4 Diffusion model-based imputation incorporating metadata information

To enhance the CSDI-based imputation method, it was modified to incorporate metadata information for each sample. Metadata variables were included during model training and excluded during evaluation. Categorical metadata variables were embedded using a FT (Gorishniy *et al.* 2021), which transforms categorical values into a *d*-dimensional representation as follows:


(7) $$\text{Embedding}=b^{\left(\text{cat}\right)}+e^{T}W^{\left(\text{cat}\right)}\in R^{d},$$

where $e^{T}$ is a one-hot vector representing the categorical values, $W^{\left(\text{cat}\right)}$ is the corresponding weight vector, and $b^{\left(\text{cat}\right)}$ is the bias term. In our experiments, the embedding dimension was set to d=8. Given *n* metadata variables, the resulting embeddings were integrated into the model using two distinct strategies:

- In the spatial concatenation approach, embeddings were appended along the feature dimension, producing input tensors of shape (C,L,K+nd).
- In the channel-wise concatenation approach, embeddings were concatenated along the channel dimension, resulting in tensors of shape (C+nd,L,K).

### 2.5 Evaluation

To evaluate imputation performance, we conducted five-fold cross-validation by randomly splitting the subjects into five groups. The MAE was calculated for the test set and used as the performance metric, defined as:


(8) $$\text{MAE}=\frac{1}{K}\Sigma_{i=1}^{K}|y_{i}-{\hat{y}}_{i}|,$$

where $y_{i}$ and ${\hat{y}}_{i}$ are observed and predicted values, respectively, and *K* is the number of bacterial features. The experimental design is illustrated in Supplementary Fig. 1, available as supplementary data at *Bioinformatics Advances* online. We compared the MAE of datasets imputed using our proposed method—the CSDI-based method with phylogenetic CNN layers (CSDI + phylum CNN)—with original CSDI-based model (standard CSDI) (Seki *et al.* 2023), linear interpolation, last observation carried forward (LOCF), and mean imputation. Each methodology is summarized in Supplementary Table 2, available as supplementary data at *Bioinformatics Advances* online.

In a downstream task to predict a binary outcome, we evaluated performance using data imputed by our proposed method, standard CSDI method, linear interpolation, and non-imputed data. To account for data imbalance, characterized by a higher proportion of negative outcomes, performance was assessed using the area under the curve (AUC) for the receiver operating characteristic (ROC) and precision-recall (PR) curves.

## 3 Results

### 3.1 Diffusion model improves imputation performance in time-series 16S rRNA data

**Figure 3.**
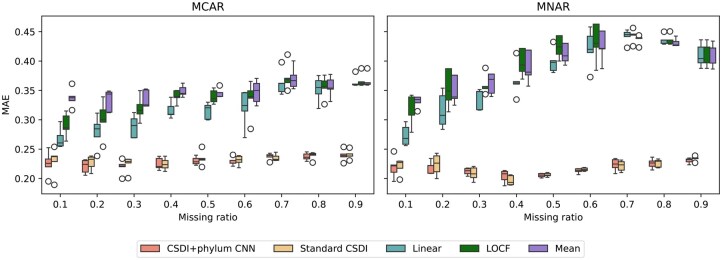
Comparison of imputation methods by MAEs of five-fold cross-validation across various missing ratios for 16S rRNA data from the DIABIMMUNE three-country cohort for two types of missing structure. The proposed CSDI method with phylum CNN consistently exhibited the lowest MAEs with low variability.

A comparative analysis of multiple methods was performed to evaluate their performance in imputing missing data in time-series 16S rRNA profiles from the three-country cohort of the DIABIMMUNE study (Vatanen *et al.* 2016). Target time points were randomly selected based on a specified missing ratio, considering two types of missing data structures: missing completely at random (MCAR) and missing not at random (MNAR). For MCAR, time points were randomly removed, whereas for MNAR, missing time points were selectively chosen based on high alpha diversity. The missing ratio *r*, defining the proportion of imputation targets, was set between 0.1 and 0.9 in increments of 0.1. The resulting mean MAEs of five-fold cross-validation across different missing ratios are presented in Table 1 and illustrated in Fig. 3. Our proposed method consistently achieves the lowest MAE scores for most of the missing ratios, suggesting that the incorporation of CNN layers may have helped suppress noise and produce more stable imputations. Notably, both CSDI-based methods showed relatively stable MAE values even as the missing ratio increased, whereas other methods exhibited a clear trend of rising errors. This is a trend that also holds for genus-level profiles, as summarized in Supplementary Table 3, available as supplementary data at *Bioinformatics Advances* online. To visualize profile similarities, we reduced the dimensionality of the imputed profiles using UMAP (McInnes *et al.* 2018) and displayed in Supplementary Fig. 2. available as supplementary data at *Bioinformatics Advances* online. Due to the differences in preprocessing pipelines, such as timepoint selection, inclusion criteria based on metadata completeness, and filtering of low-abundance features, the scores reported in Table 1 differ from those from previous studies (Choi *et al.* 2023, Seki *et al.* 2023). For reference, we also evaluated our method using preprocessed datasets from previous studies and found that it consistently outperformed other methods as summarized in Supplementary Table 4, available as supplementary data at *Bioinformatics Advances* online.

**Table 1.** Comparison of MAEs from five-fold cross-validation across various missing ratios for 16S rRNA data from the DIABIMMUNE study for two types of missing data structures: MCAR and MNAR.^a^

Missing structure	Missing ratio	CSDI + phylum CNN	Standard CSDI	Linear interpolation	LOCF	Mean
MCAR	0.1	**0.225** (0.021)	0.230 (0.024)	0.268 (0.018)	0.291 (0.020)	0.338 (0.016)
	0.2	**0.221** (0.012)	0.227 (0.013)	0.280 (0.027)	0.301 (0.031)	0.333 (0.019)
	0.3	**0.220** (0.012)	0.224 (0.014)	0.289 (0.019)	0.319 (0.021)	0.335 (0.015)
	0.4	**0.225** (0.010)	**0.225** (0.010)	0.316 (0.014)	0.340 (0.011)	0.349 (0.010)
	0.5	**0.230** (0.007)	0.234 (0.012)	0.315 (0.014)	0.339 (0.012)	0.346 (0.008)
	0.6	**0.229** (0.007)	0.231 (0.008)	0.320 (0.031)	0.335 (0.030)	0.347 (0.020)
	0.7	0.237 (0.006)	**0.235** (0.006)	0.363 (0.021)	0.373 (0.023)	0.371 (0.019)
	0.8	**0.237** (0.006)	0.239 (0.007)	0.352 (0.022)	0.356 (0.019)	0.356 (0.018)
	0.9	**0.240** (0.010)	0.240 (0.008)	0.364 (0.010)	0.367 (0.012)	0.366 (0.012)
**MNAR**	0.1	**0.219** (0.019)	0.220 (0.013)	0.273 (0.018)	0.318 (0.026)	0.326 (0.011)
	0.2	**0.219** (0.011)	0.224 (0.018)	0.316 (0.030)	0.357 (0.037)	0.348 (0.021)
	0.3	0.212 (0.006)	**0.208** (0.011)	0.336 (0.018)	0.363 (0.018)	0.362 (0.018)
	0.4	0.204 (0.011)	**0.197** (0.008)	0.367 (0.029)	0.392 (0.019)	0.384 (0.022)
	0.5	**0.205** (0.003)	0.206 (0.003)	0.399 (0.021)	0.416 (0.018)	0.407 (0.014)
	0.6	**0.213** (0.004)	0.216 (0.003)	0.422 (0.032)	0.430 (0.030)	0.424 (0.024)
	0.7	0.224 (0.010)	**0.222** (0.009)	0.442 (0.012)	0.445 (0.012)	0.439 (0.010)
	0.8	**0.226** (0.008)	0.226 (0.007)	0.436 (0.008)	0.437 (0.009)	0.432 (0.006)
	0.9	**0.230** (0.005)	0.234 (0.004)	0.410 (0.020)	0.410 (0.020)	0.409 (0.019)

a Each cell represents mean (SD). Bold font represents the best score.

The human gut holds highly diverse bacterial profiles between hosts, resulting in the sparsity. To assess if imputed profiles reflect these characteristics, we used two indices: alpha diversity and zero abundance proportion. Alpha diversity measures the variety within a single sample typically assessed using the Shannon index. As shown in Supplementary Fig. 3, available as supplementary data at *Bioinformatics Advances* online, alpha diversity rises over time, stabilizing as infants develop and acquire microbiota diversity. Imputed profiles by our method follow this trend over time, while linear interpolation shows a weak trend with a wider range. Our proposed method also captures the decreasing trend of the proportions of zero abundance illustrated in Supplementary Fig. 4, available as supplementary data at *Bioinformatics Advances* online.

The MAEs calculated by each bacterial species were visualized in Supplementary Fig. 5, available as supplementary data at *Bioinformatics Advances* online and the bacterial species with the highest and lowest MAEs are listed in Supplementary Fig. 6, available as supplementary data at *Bioinformatics Advances* online. We observed substantial variation in MAE magnitudes across species, particularly among those with high MAEs. We plotted the time courses of these bacterial species in Supplementary Fig. 7, available as supplementary data at *Bioinformatics Advances* online to further examine this variability. Predicting species with low abundance levels is generally more straightforward, as imputations of low values yield smaller MAEs, while predicting species with higher abundances is more challenging. Supplementary Figure 7a, available as supplementary data at *Bioinformatics Advances* online, demonstrates, however, that our method successfully captures the overall temporal trends in bacterial abundances. Of note, “s__unclassified” appears in the species, indicating that unclassified species are grouped together and present as high-abundance bacteria. This aggregation complicates accurate prediction, and improved bacterial annotation likely enhances prediction performance.

### 3.2 Downstream predictive task can be improved using profiles imputed by proposed method

We conducted a downstream predictive analysis using a bidirectional RNN-based model, the architecture of which is illustrated in Supplementary Fig. 8, available as supplementary data at *Bioinformatics Advances* online. This model processes time-series inputs recursively and is followed by a dense layer for disease presence classification. To adequately train the prediction model, we generated 300 simulated samples from the 16S rRNA profiles of the DIABIMMUNE three-country cohort (Vatanen *et al.* 2016). The 116 subjects were split into training and test set by 7:3 ratio. Within each set, 210 and 90 subjects, respectively, were sampled with replication. Following the method used by Sharma and Xu (2021), normally distributed noise with a randomly selected mean from the range ($1\times 10^{-4},2\times 10^{-4}$) and a standard deviation of $10^{-4}$ was added. The relative abundances were adjusted to ensure they summed to 1 by following the approach used in previous work (Choi *et al.* 2023), where the small difference due to this zero-setting operation is partitioned by the number of nonzero species and added to each of the nonzero species. The subject’s health status to be predicted were derived from metadata information such as milk allergy (simulation #1), egg allergy (simulation #2), and peanut allergy (simulation #3). Each variable is binary with various positive proportions as summarized in Supplementary Table 5, available as supplementary data at *Bioinformatics Advances* online. For the simulated test set of 90 subjects, missing time points were randomly selected and then imputed using one of the following methods: our CSDI method with phylum CNN, standard CSDI, or linear interpolation. Prediction was then performed using a combined dataset consisting of 210 training subjects (with no missing time points) and the 90 imputed test subjects. We also performed prediction using the incomplete dataset before imputation with masking strategy which accommodates the dataset’s heterogeneous lengths. The original complete dataset was also used as a reference to establish a baseline.

**Figure 4.**
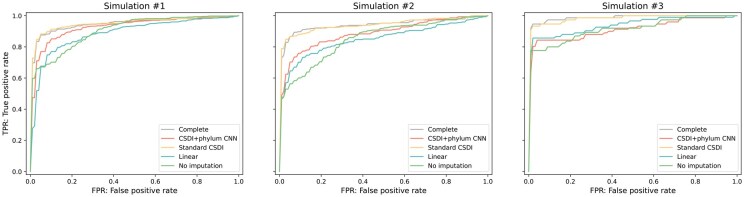
Mean ROC curves from 5× five-fold cross-validation on the simulated 16S rRNA dataset. The proposed method achieves performance comparable with that of the complete data and outperformed existing methods, particularly in Simulations #1 and #2.

The mean ROC curves are visualized in Fig. 4 and each ROC-AUC and PR-AUC value is summarized in Supplementary Table 6, available as supplementary data at *Bioinformatics Advances* online. To ensure the robustness of our results and account for variability, we performed 5× five-fold cross-validations with different splits into training and test sets. Among the imputation methods, the standard CSDI model (Seki *et al.* 2023) achieved the highest predictive performance, closely matching that of the complete dataset. Our proposed CNN-augmented method outperformed both linear interpolation and the nonimputed dataset, particularly in Simulations #1 and #2. This consistent performance suggests that the profiles imputed by our method retain biologically relevant signals present in the true profiles, enabling predictive models to effectively capture associations with host health, even when the data is incomplete. In Simulation #3, which involved an extremely imbalanced outcome (94% negative samples), the CNN-enhanced model showed relatively poor predictive performance, comparable with that of linear interpolation and the nonimputed dataset. This may reflect a trade-off introduced by the CNN layers: while they effectively suppress noise and stabilize imputation under typical conditions, they may also attenuate subtle signal variations that are important in highly imbalanced scenarios. These findings indicate that there is room to further refine architectural choices, such as the integration of CNN layers, based on the characteristics of specific prediction tasks.

### 3.3 Incorporating metadata information in proposed imputation method

To advance imputation performance, we hypothesized that incorporating external metadata could enhance the model’s robustness and accuracy, given the significant variability in bacterial composition among individuals. Inspired by CSDI_T (Zheng and Charoenphakdee 2022), a CSDI model designed for tabular data, we modified the CSDI model with CNN to embed and concatenate categorical metadata with abundance profiles. In the DIABIMMUNE study, it was noted that country, alongside age, was identified as a major source of variation, particularly during the first year of life (Vatanen *et al.* 2016). Additionally, allergy information (milk, egg, and peanut) has demonstrated predictive potential based on previous studies using this dataset (Sharma and Xu 2021, Choi *et al.* 2023) and our findings. To investigate the differentiability of those metadata variables, PERMANOVA analysis (Anderson 2017) was performed and the principal coordinate analysis (PCoA) plots at each time point are presented in Supplementary Figs 9–12, available as supplementary data at *Bioinformatics Advances* online. The profiles generally overlap, but some differences are observed especially in country, egg allergy at later time points, and peanut allergy at earlier time points, indicating that these metadata potentially contribute to generating imputed profiles. We conducted training using metadata for country and allergy presence, using two distinct strategies explained in the Methods section.

**Figure 5.**
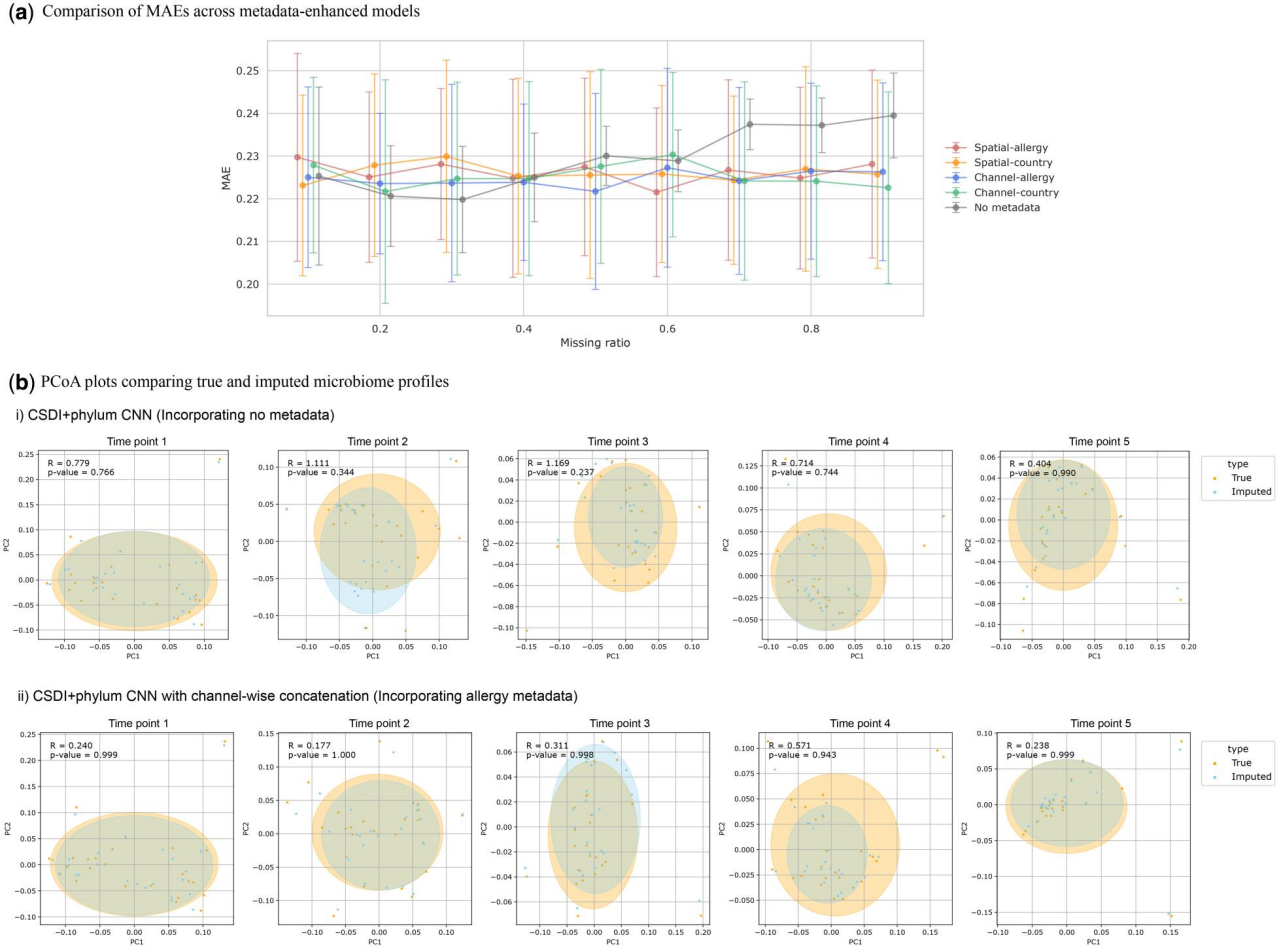
Performance of metadata-enhanced CSDI models. (a) Comparison of MAEs across models incorporating metadata information by five-fold cross-validation for 16S rRNA data from the DIABIMMUNE three-country cohort. (b) PCoA plots comparing true and imputed profiles. Missing values were imputed by (i) the original CSDI + phylum CNN model, and (ii) the metadata-enhanced CSDI model. Greater overlap with the true profiles is observed in the metadata-enhanced model, indicating improved reconstruction with metadata integration.

The results in Fig. 5a show that the modified model achieved measurable improvements across missing ratios, particularly when the missing ratio exceeded 0.4. While the observed improvements were modest, they present the feasibility of integrating metadata to enhance imputation performance. No substantial difference was observed between the two embedding concatenation directions, although channel-wise concatenation of allergy metadata tended to produce more stable results. To further assess the impact of metadata integration, Fig. 5b displays PCoA plots comparing profiles imputed by the original model and the metadata-enhanced model utilizing allergy information under a missing ratio of 0.5. The imputed profiles from the metadata-informed model show greater overlap with the true profiles, suggesting improved reconstruction fidelity. While the gains remain limited, potentially due to the metadata’s moderate discriminative power and the constraints of the embedding method, these findings underscore the promise of metadata integration in microbiome imputation and highlight the need to identify and incorporate more informative metadata in future research.

### 3.4 Diffusion model-based method presents superiority in imputing time-series WGS data

In addition to the 16S rRNA dataset, we evaluated the imputation performance for WGS datasets obtained from the BONUS study (Hayden *et al.* 2020). WGS sequencing is another powerful resource for investigating microbiome composition and functionality. Given the higher cost of WGS sequencing, effectively obtaining complete datasets from partial data is particularly valuable. As metadata information was not publicly available for the BONUS dataset, we conducted this experiment using the CSDI model with phylum CNN layers, without metadata integration.

**Figure 6.**
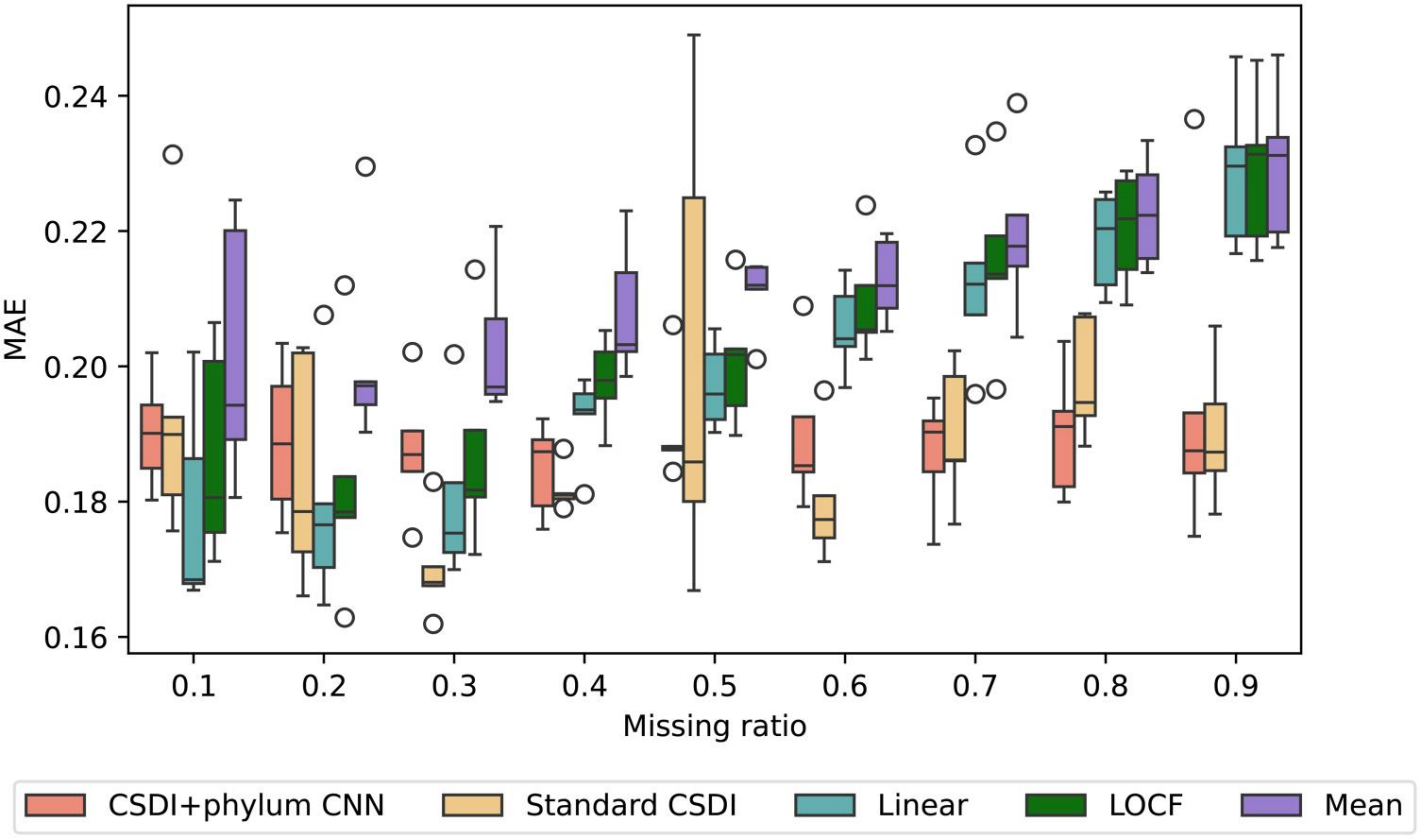
Comparison of imputation methods by MAEs of five-fold cross-validation across various missing ratios for WGS data from the BONUS study. The proposed CSDI method with CNN consistently exhibited low MAEs especially at higher missing ratios.

The CSDI-based methods demonstrate stable MAEs compared with other methods across most missing ratios, as shown in Table 2 and Fig. 6. At missing ratios of 0.1 and 0.2, slightly higher mean MAE values were observed across folds, but no unusual patterns were identified in the UMAP visualization (Supplementary Fig. 13, available as supplementary data at *Bioinformatics Advances* online). One possible explanation is that complex models can introduce greater bias under low missing ratios in zero-inflated datasets. However, in almost every case, our method consistently achieved low MAEs, demonstrating its robustness in handling the complexity of WGS datasets, which contain more than 7× the number of features compared with 16S rRNA data. The scalability of our approach across both 16S rRNA and WGS datasets highlights its versatility and potential for application in larger, more diverse microbiome studies. Analysis of the imputed profiles (Supplementary Figs 14 and 15, available as supplementary data at *Bioinformatics Advances* online) revealed that the method tended to predict higher diversity and faced challenges in accurately predicting zero abundance. These challenges likely stem from the high dimensionality of WGS profiles, and addressing this limitation will be a key focus for future refinement. Despite these challenges, the method’s ability to handle diverse datasets and its potential for broader applications underscore its value for advancing microbiome research.

**Table 2.** Comparison of imputation methods by mean MAEs of five-fold cross-validation for WGS data from BONUS study.^a^

Missing ratio	CSDI + phylum CNN	Standard CSDI	Linear interpolation	LOCF	Mean
0.1	0.190 (0.008)	0.194 (0.022)	**0.178 (0.016)**	0.187 (0.016)	0.202 (0.019)
0.2	0.189 (0.012)	0.184 (0.017)	**0.180 (0.017)**	0.183 (0.018)	0.202 (0.016)
0.3	0.188 (0.010)	**0.170 (0.008)**	0.180 (0.013)	0.188 (0.016)	0.203 (0.011)
0.4	0.185 (0.007)	**0.182 (0.003)**	0.192 (0.007)	0.198 (0.007)	0.208 (0.010)
0.5	**0.191 (0.009)**	0.201 (0.034)	0.197 (0.006)	0.201 (0.010)	0.211 (0.006)
0.6	0.190 (0.012)	**0.180 (0.010)**	0.206 (0.007)	0.209 (0.009)	0.213 (0.006)
0.7	**0.187 (0.008)**	0.190 (0.010)	0.213 (0.013)	0.215 (0.014)	0.220 (0.013)
0.8	**0.190 (0.010)**	0.198 (0.009)	0.218 (0.007)	0.220 (0.008)	0.223 (0.008)
0.9	0.195 (0.024)	**0.190 (0.011)**	0.229 (0.012)	0.229 (0.012)	0.230 (0.012)

a Each cell represents mean (SD). Bold font represents the best score.

## 4 Discussion

In this study, we proposed an imputation method for time-series microbiota datasets based on the diffusion model framework, CSDI (Tashiro *et al.* 2021). To adapt the method for microbial data, we modified the denoising function to incorporate phylogenetic information using CNNs for each phylum category. Our method consistently achieved lower MAEs across most missing ratios in 16S rRNA datasets and competitive performance in WGS datasets, highlighting its stable and robust performance. The incorporation of CNN layers for phylum categories likely helped reduce noise arising from measurement variability and preprocessing, thereby contributing to improved overall imputation accuracy. The imputed profiles tended to preserve key characteristics of realistic microbial data, including alpha diversity and zero-abundance features, though this preservation may vary depending on data type and missing rate. Capturing these features accurately is crucial for identifying microbial patterns linked to host status. In downstream predictive tasks, the performance of the datasets imputed by our method was comparable with that of the original complete datasets. These findings indicate that our imputation method reliably supports downstream analyses and enables the exploration of microbiome associations with health-related factors, such as disease presence in longitudinal studies. We also introduced a metadata-integrated version of the CSDI model, which incorporates host-related information such as allergy status. This extension showed strong potential to improve imputation performance, particularly under higher missing ratios.

However, there are limitations to our current approach. While our proposed method reduced MAE across various missing ratios and patterns, its benefit in downstream prediction tasks was limited compared with the standard CSDI model. This may be attributed to the CNN layers, which effectively suppress noise and reduce reconstruction error, but may also attenuate significant fluctuations in microbial abundances. This trade-off between denoising and preserving informative variation warrants further investigation and may need to be tuned according to the specific goals of downstream analysis. In addition, while our method effectively captures the overall temporal trends in bacterial abundances, errors persist, particularly for bacteria with high abundance. To further refine the method, we explored the inclusion of metadata. This led to modest improvements in MAE, possibly due to architectural changes whereby metadata variables were leveraged as conditional inputs during denoising. The limited gains may reflect the limited influence of background metadata on time-series compositional patterns. Nonetheless, incorporating external contextual information remains a promising direction. If external factors can be successfully integrated into the imputation process, the approach could potentially extend beyond simply reconstructing missing values to predicting the effects of interventions, such as medications or dietary changes, on microbiota composition.

In summary, our proposed imputation method demonstrates strong performance in addressing missing data in time-series microbiota profiles. Future research should focus on improving sensitivity and effectively incorporating external metadata to enhance the method’s utility and reliability. By overcoming these challenges, this approach has the potential to advance our understanding of microbiota-host interactions and contribute to the development of better predictive models for health outcomes.

## 5 Conclusion

We proposed a diffusion model-based imputation method for time-series microbiome profiles. Our method effectively reduces MAE across various missing data ratios and improved the prediction of host health status compared with existing methods. Furthermore, incorporating metadata showed potential to further enhance imputation performance. This work provides a practical solution for leveraging incomplete time-series microbial data in longitudinal studies by reliably imputing missing profiles.

## Supplementary Material

vbaf181_Supplementary_Data
