# Supplementary material for: Diffusion model for imputing time-series gut microbiome profiles using phylogenetic information and metadata integration
Source: Bioinform Adv. 2025 Jul 28;5(1):vbaf181. doi: 10.1093/bioadv/vbaf181 (PMC12371328; doi:10.1093/bioadv/vbaf181)
Supplement: vbaf181_Supplementary_Data [file vbaf181_supplementary_data.pdf]

Diffusion model for imputing time-series gut microbiome profiles using phylogenetic information and metadata integration

Supplementary data

List of Tables

Supplementary Table 1 Summary of dataset used in the experiments of missing time point imputation. . . . 3
Supplementary Table 2 Summary of each imputation method used in the comparative analysis. . . . 3
Supplementary Table 3 Comparison of imputation methods by mean MAEs of 5-fold cross-validation across various missing ratios for 16S rRNA data at the genus level. . . . 4
Supplementary Table 4 Transposed comparison of mean MAEs from 5-fold cross-validation across various missing ratios for 16S rRNA data from DIABIMMUNE study. Bold font represents the best score. . . . 4
Supplementary Table 5 Distribution of the values for each outcome included in the simulated 16S rRNA dataset derived from the DIABIMMUNE three country cohort. . . . 4
Supplementary Table 6 Comparison of mean ROC-AUC and PR-AUC by five-time 5-fold cross-validation on simulated 16S rRNA dataset . . . 4

List of Figures

Supplementary Fig. 1 Experimental design. . . . 5
Supplementary Fig. 2 UMAP plots of imputed profiles for the first fold of 5-fold cross-validation for 16S rRNA data from the DIABIMMUNE three-country cohort. . . . 6
Supplementary Fig. 3 Distribution of alpha diversity by time point across various missing ratios for 16S rRNA data from DIABIMMUNE three country cohort. 7
Supplementary Fig. 4 Distribution of proportion of zero abundance by time point across various missing ratios for 16S rRNA data from DIABIMMUNE three country cohort. . . . 7
Supplementary Fig. 5 Distribution of MAEs calculated for each bacteria species in the imputed 16S rRNA profile from the DIABIMMUNE study with missing ratio of 0.5. . . . 8
Supplementary Fig. 6 The individual names and MAEs of bacteria which have (a) top 10 largest MAE and (b) bottom 10 smallest MAE . . . 8
Supplementary Fig. 7 Time-course plot for each bacteria which shows (a) top 10 largest MAE and (b) bottom 10 smallest MAE . . . 9
Supplementary Fig. 8 Architecture of the bidirectional RNN-based model used to predict disease presence. . . . 9
Supplementary Fig. 9 PCoA plots with PERMANOVA results on 16S rRNA profile from the DIABIMMUNE study for country. . . . 10
Supplementary Fig. 10 PCoA plots with PERMANOVA results on 16S rRNA profile from the DIABIMMUNE study for presence of milk allergy. . . . 10
Supplementary Fig. 11 PCoA plots with PERMANOVA results on 16S rRNA profile from the DIABIMMUNE study for presence of egg allergy. . . . 11
Supplementary Fig. 12 PCoA plots with PERMANOVA results on 16S rRNA profile from the DIABIMMUNE study for presence of peanut allergy. . . . 11
Supplementary Fig. 13 UMAP plots of imputed profiles for the first fold out of 5-fold cross-validation for WGS data from BONUS study. . . . 12
Supplementary Fig. 14 Distribution of alpha diversity by time point across various missing ratios for WGS data from BONUS study. . . . 13

Supplementary Fig. 15 Distribution of proportion of zero abundance by time point across various missing ratios for WGS data from BONUS study. 13

Supplementary Table 1: Summary of dataset used in the experiments of missing time point imputation. mo: months old.

Study name	DIABIMMUNE [1]	BONUS [2]
Sequencing type	16S rRNA	WGS
Length of time points	5	7
Time point names	0, 6, 12, 18, and 24 months	3, 4, 5, 6, 8, 10, and 12 months
Interval of time points	6 months	1-2 months
Number of subjects	115	157
Number of total samples	580	975
Number of bacteria species	113	833
Age	0 to 24 mo	3 to 12 mo
Female (%)	45.7	50.3
Country	Estonia, Finland, Russia	US

Supplementary Table 2: Summary of each imputation method used in the comparative analysis.

Methods	Details
CSDI+phylum CNN	Our proposed imputation method using a diffusion model framework for time-series microbiome data, incorporating CNN layers for phylum categories.
Standard CSDI	An original imputation method using a diffusion model framework for time-series microbiome data proposed in [3].
Linear interpolation	An imputation method that applies linear interpolation between known previous and subsequent data points to estimate missing values.
LOCF	An imputation method that fills in missing data by carrying forward the last valid observation. In cases with no prior valid observations, the next valid observation is used in our experiment.
Mean	An imputation method that fills in missing values with the mean of observed values for each subject.

Supplementary Table 3: Comparison of imputation methods by mean MAEs of 5-fold cross-validation across various missing ratios for 16S rRNA data at the genus level. Bold font represents the best score.

Missing Ratio	CSDI+phylum CNN	Standard CSDI	Linear interpolation	LOCF	Mean
0.1	**0.270** (0.031)	**0.270** (0.033)	0.308 (0.018)	0.340 (0.021)	0.386 (0.017)
0.2	**0.266** (0.016)	0.270 (0.020)	0.323 (0.036)	0.363 (0.043)	0.376 (0.027)
0.3	**0.269** (0.011)	0.272 (0.015)	0.345 (0.020)	0.387 (0.021)	0.388 (0.012)
0.4	**0.267** (0.015)	**0.267** (0.010)	0.373 (0.015)	0.405 (0.011)	0.403 (0.009)
0.5	**0.267** (0.007)	0.274 (0.011)	0.378 (0.020)	0.407 (0.026)	0.408 (0.007)
0.6	**0.280** (0.008)	0.284 (0.012)	0.377 (0.045)	0.395 (0.040)	0.406 (0.031)
0.7	**0.282** (0.011)	0.286 (0.011)	0.420 (0.027)	0.434 (0.029)	0.429 (0.023)
0.8	0.283 (0.011)	**0.278** (0.015)	0.407 (0.015)	0.413 (0.008)	0.412 (0.012)
0.9	**0.284** (0.011)	0.287 (0.011)	0.424 (0.010)	0.427 (0.011)	0.425 (0.009)

Supplementary Table 4: Transposed comparison of mean MAEs from 5-fold cross-validation across various missing ratios for 16S rRNA data from DIABIMMUNE study. Bold font represents the best score.

Missing Ratio	CSDI+phylum CNN	DeepMicroGen	Linear interpolation	LOCF	Mean
0.1	**1.563** (0.075)	-	1.737 (0.074)	1.931 (0.089)	1.835 (0.100)
0.2	**1.610** (0.090)	-	1.758 (0.147)	1.939 (0.146)	1.810 (0.060)
0.3	**1.559** (0.063)	1.573	1.788 (0.083)	2.051 (0.090)	1.811 (0.075)
0.4	**1.587** (0.074)	1.589	1.840 (0.117)	2.050 (0.072)	1.844 (0.070)
0.5	**1.582** (0.025)	1.616	1.966 (0.136)	2.143 (0.103)	1.922 (0.068)
0.6	**1.592** (0.051)	1.642	2.019 (0.091)	2.172 (0.070)	1.966 (0.076)
0.7	**1.630** (0.017)	1.741	2.078 (0.092)	2.212 (0.059)	2.053 (0.081)
0.8	**1.609** (0.033)	-	2.160 (0.087)	2.242 (0.035)	2.136 (0.085)
0.9	**1.614** (0.033)	-	2.259 (0.085)	2.280 (0.084)	2.254 (0.071)

Supplementary Table 5: Distribution of the values for each outcome included in the simulated 16S rRNA dataset derived from the DIABIMMUNE three country cohort. Outcome #1 is derived from milk allergy, Outcome #2 is derived from egg allergy and Outcome #3 is derived from peanut allergy.

Outcome	Positive	Negative
#1	96 (32%)	203 (68%)
#2	58 (19%)	241 (80%)
#3	16 (5%)	283 (94%)

Supplementary Table 6: Comparison of mean ROC-AUC and PR-AUC by five-time 5-fold cross-validation on simulated 16S rRNA dataset across different imputation methods: original complete data, proposed CSDI method with phylum, standard CSDI method, Linear interpolation and No imputation.

	Simulation	Complete data (Reference)	CSDI+phylum CNN	Standard CSDI	Linear	No imputation
ROC-AUC	#1	0.948 (0.065)	0.938 (0.042)	0.942 (0.063)	0.921 (0.058)	0.908 (0.034)
	#2	0.959 (0.035)	0.906 (0.076)	0.958 (0.038)	0.936 (0.052)	0.858 (0.069)
	#3	0.982 (0.040)	0.961 (0.082)	0.979 (0.046)	0.961 (0.063)	0.927 (0.094)
PR-AUC	#1	0.934 (0.063)	0.919 (0.055)	0.930 (0.062)	0.891 (0.075)	0.869 (0.038)
	#2	0.932 (0.044)	0.859 (0.102)	0.926 (0.046)	0.883 (0.068)	0.725 (0.115)
	#3	0.907 (0.145)	0.915 (0.126)	0.925 (0.128)	0.865 (0.157)	0.784 (0.215)

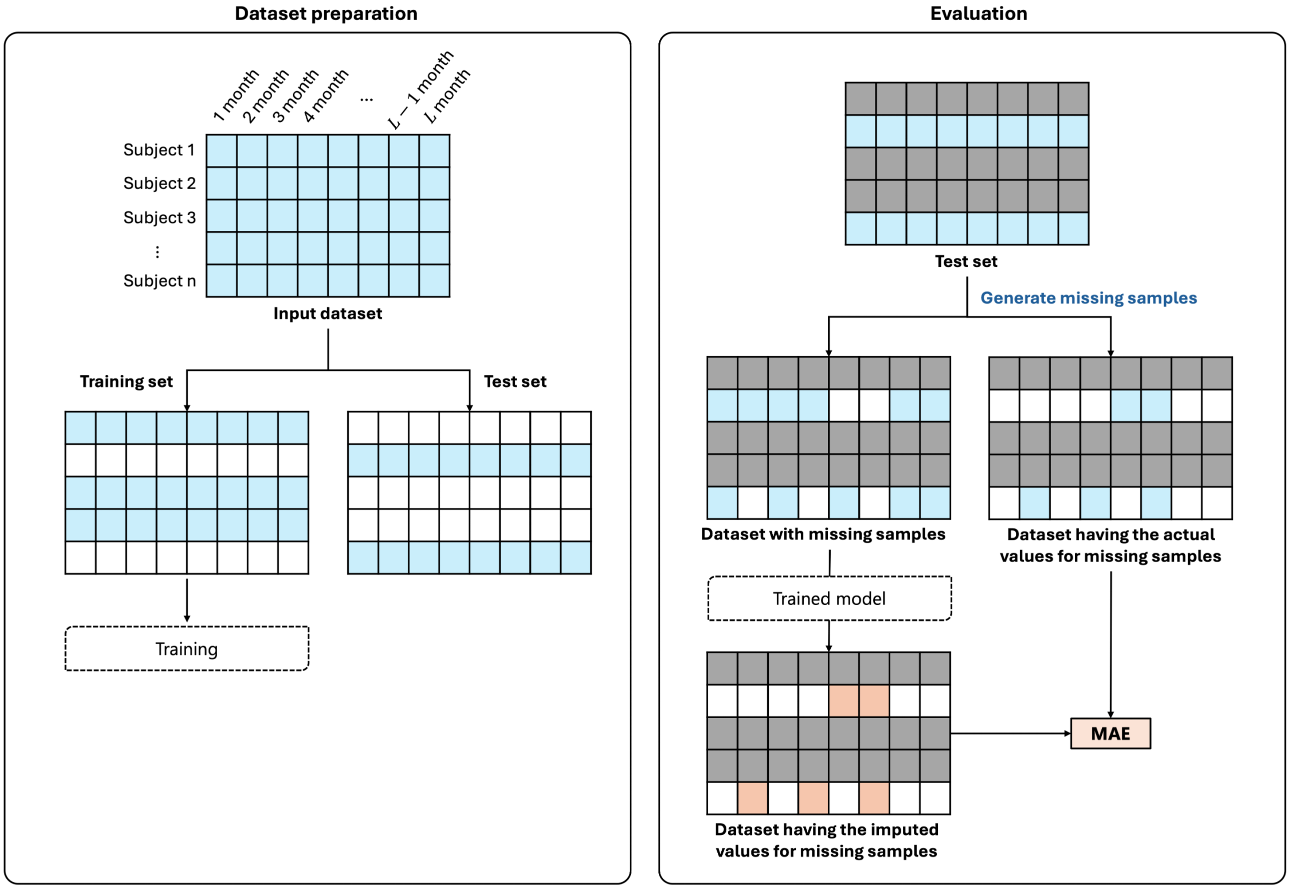

Supplementary Fig. 1: The experimental design. The input dataset is first divided into training and test sets based on subject IDs. Missing time points are then artificially selected in the test set and imputed using the trained model. Mean absolute error (MAE) is calculated by comparing the imputed values to the corresponding ground truth.

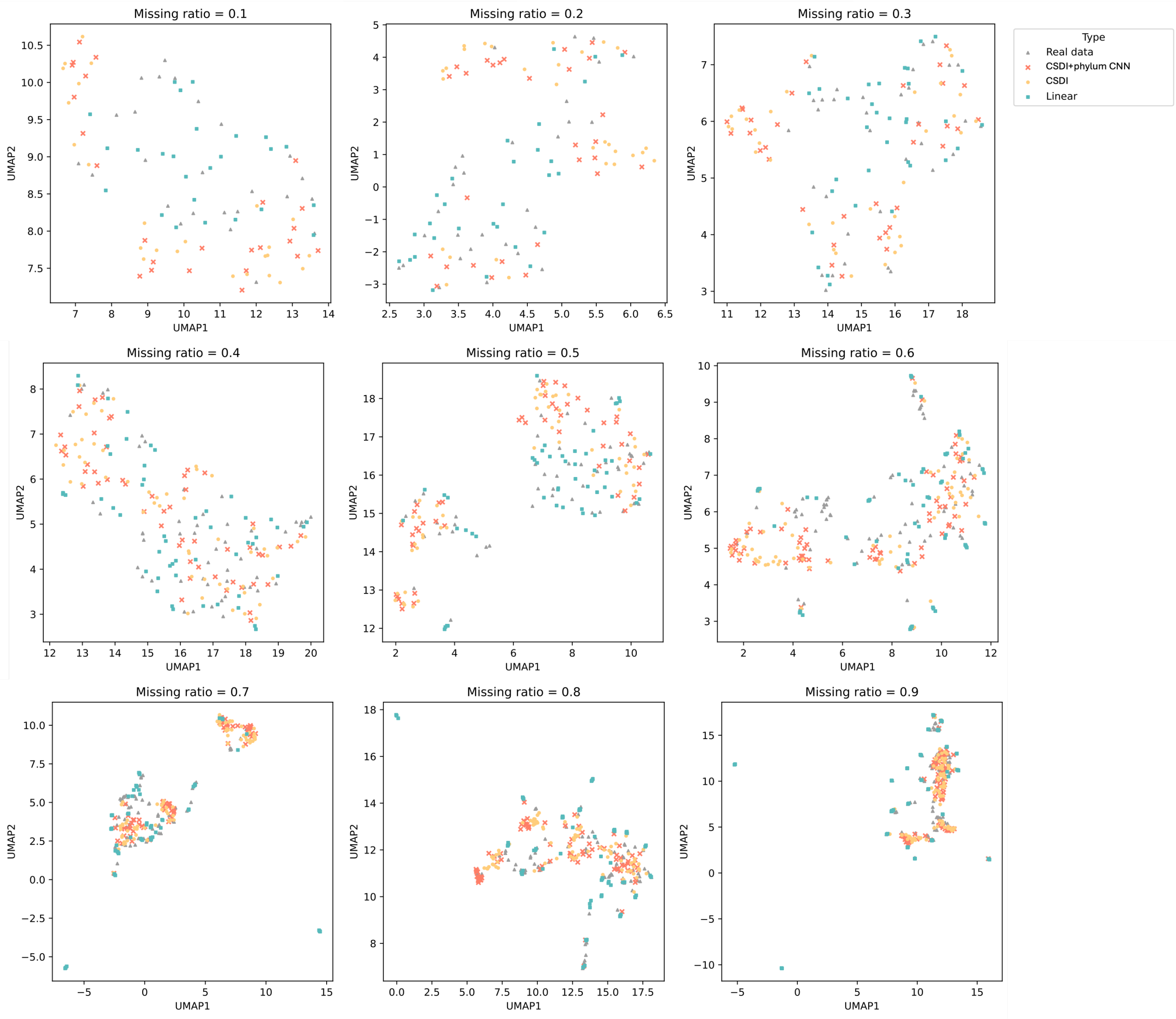

Supplementary Fig. 2: UMAP plots of imputed profiles for the first fold of 5-fold cross-validation for 16S rRNA data from the DIABIMMUNE three-country cohort.

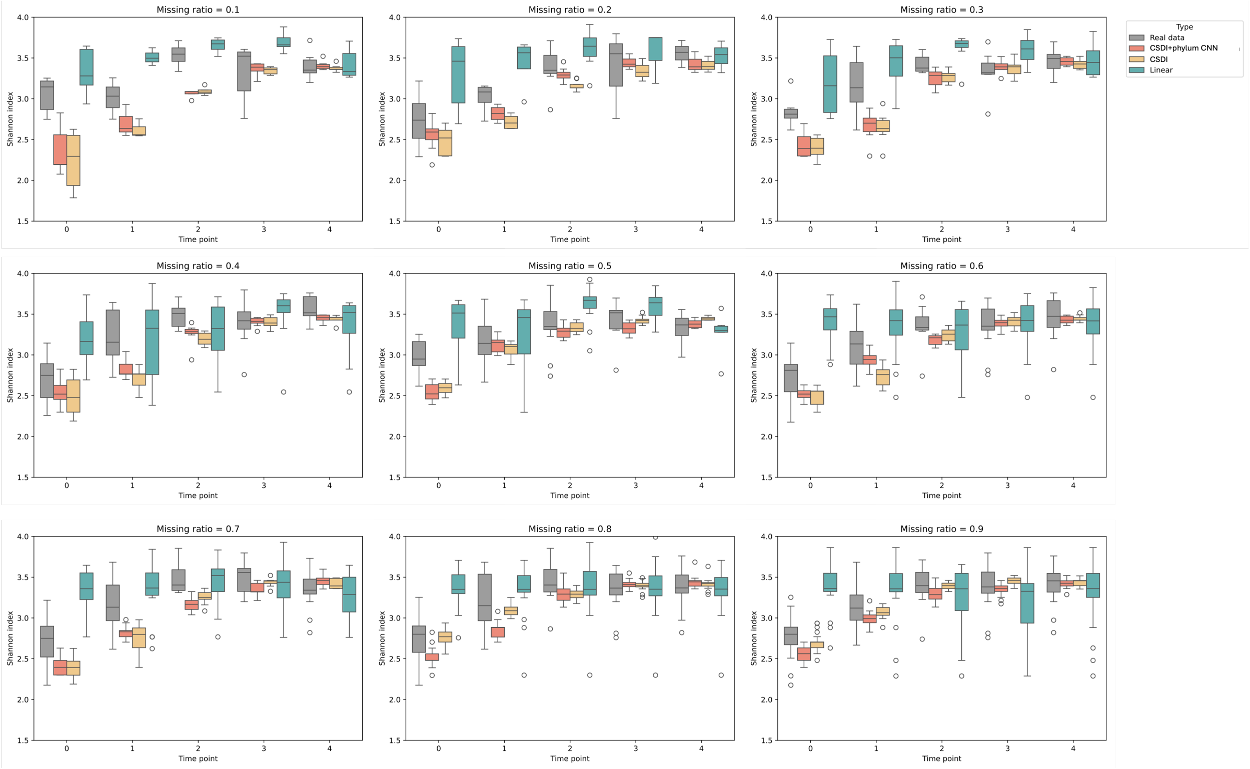

Supplementary Fig. 3: Distribution of **alpha diversity** by time point across various missing ratios for 16S rRNA data from DIABIMMUNE three country cohort.

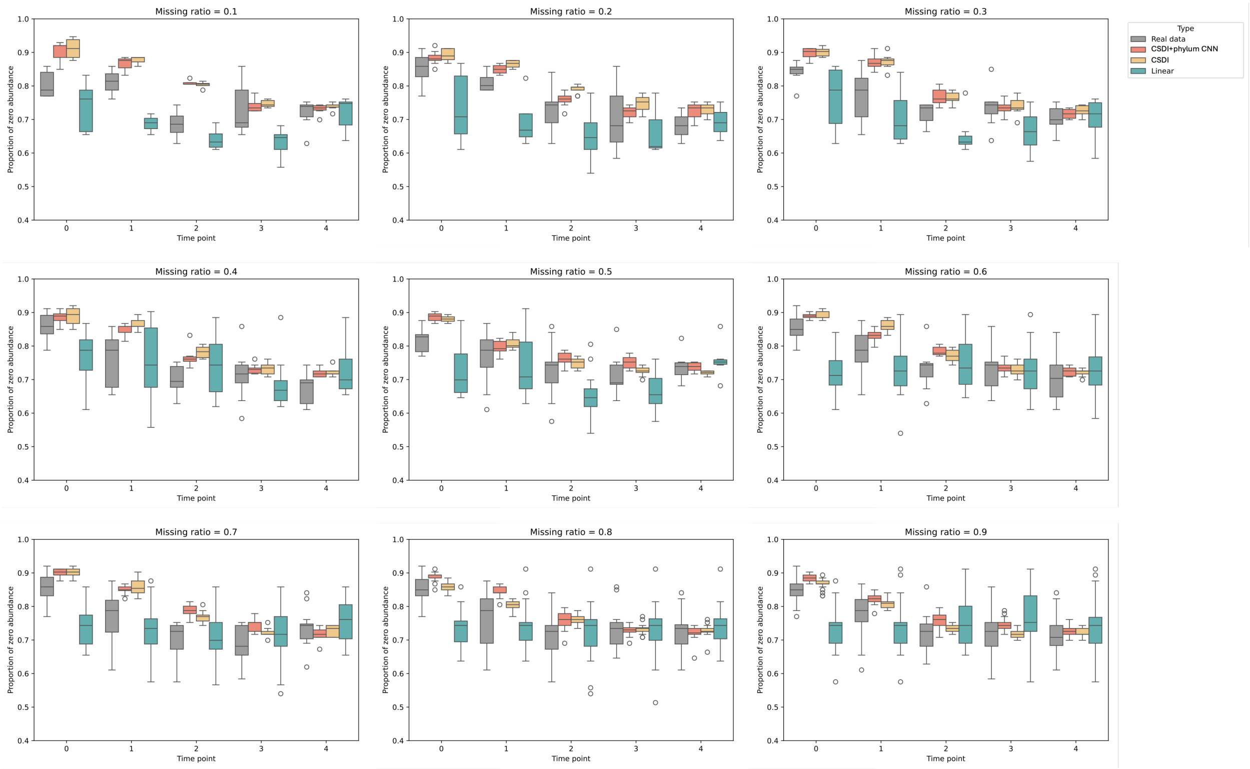

Supplementary Fig. 4: Distribution of **proportion of zero abundance** by time point across various missing ratios for 16S rRNA data from DIABIMMUNE three country cohort.

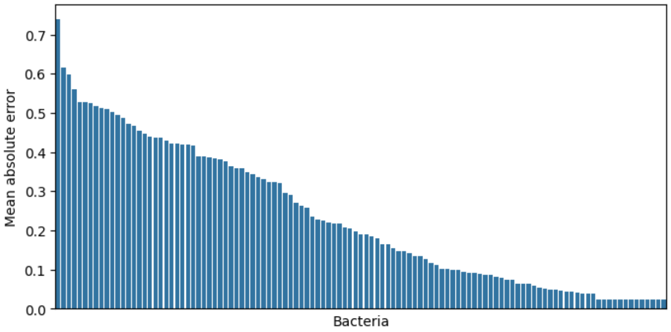

Supplementary Fig. 5: Distribution of MAEs calculated for each bacteria species in the imputed 16S rRNA profile from the DIABIMMUNE study with missing ratio of 0.5.

(a) Top 10 bacteria with large MAE

<table>
<tr><th>Bacteria</th><th>MAE</th></tr>
<tr><td>k__Bacteria|p__Firmicutes|c__Clostridia|o__Clostridiales|f__Veillonellaceae|g__Veillonella|s__parvula</td><td>0.739</td></tr>
<tr><td>k__Bacteria|p__Proteobacteria|c__Gammaproteobacteria|o__Pasteurellales|f__Pasteurellaceae|g__Haemophilus|s__unclassified</td><td>0.615</td></tr>
<tr><td>k__Bacteria|p__Firmicutes|c__Clostridia|o__Clostridiales|f__Veillonellaceae|g__Veillonella|s__dispar</td><td>0.598</td></tr>
<tr><td>k__Bacteria|p__Firmicutes|c__Clostridia|o__Clostridiales|f__Veillonellaceae|g__unclassified|s__unclassified</td><td>0.561</td></tr>
<tr><td>k__Bacteria|p__Bacteroidetes|c__Bacteroidia|o__Bacteroidales|f__Bacteroidaceae|g__Bacteroides|s__unclassified</td><td>0.528</td></tr>
<tr><td>k__Bacteria|p__Firmicutes|c__Clostridia|o__Clostridiales|f__Lachnospiraceae|g__Lachnospira|s__unclassified</td><td>0.527</td></tr>
<tr><td>k__Bacteria|p__Firmicutes|c__Bacilli|o__Lactobacillales|f__Carnobacteriaceae|g__Granulicatella|s__unclassified</td><td>0.527</td></tr>
<tr><td>k__Bacteria|p__Firmicutes|c__Clostridia|o__Clostridiales|f__Lachnospiraceae|g__[Ruminococcus]|s__gnavus</td><td>0.517</td></tr>
<tr><td>k__Bacteria|p__Proteobacteria|c__Betaproteobacteria|o__Burkholderiales|f__Alcaligenaceae|g__Sutterella|s__unclassified</td><td>0.513</td></tr>
<tr><td>k__Bacteria|p__Verrucomicrobia|c__Verrucomicrobiae|o__Verrucomicrobiales|f__Verrucomicrobiaceae|g__Akkermansia|s__muciniphila</td><td>0.509</td></tr>
</table>

(b) Bottom 10 bacteria with small MAE

<table>
<tr><th>Bacteria</th><th>MAE</th></tr>
<tr><td>k__Bacteria|p__Bacteroidetes|c__Bacteroidia|o__Bacteroidales|f__Prevotellaceae|g__Prevotella|s__stercorea</td><td>0.0234</td></tr>
<tr><td>k__Bacteria|p__Firmicutes|c__Bacilli|o__Lactobacillales|f__Lactobacillaceae|g__unclassified|s__unclassified</td><td>0.0234</td></tr>
<tr><td>k__Bacteria|p__Firmicutes|c__Clostridia|o__Clostridiales|f__Lachnospiraceae|g__Blautia|s__obeum</td><td>0.0234</td></tr>
<tr><td>k__Bacteria|p__Firmicutes|c__Clostridia|o__Clostridiales|f__Clostridiaceae|g__Peptoniphilus|s__asaccharolyticus</td><td>0.0234</td></tr>
<tr><td>k__Bacteria|p__Proteobacteria|c__Gammaproteobacteria|o__Enterobacteriales|f__Enterobacteriaceae|g__Trabulsiella|s__unclassified</td><td>0.0234</td></tr>
<tr><td>k__Bacteria|p__Actinobacteria|c__Actinobacteria|o__Actinomycetales|f__Corynebacteriaceae|g__Corynebacterium|s__unclassified</td><td>0.0234</td></tr>
<tr><td>k__Bacteria|p__Bacteroidetes|c__Bacteroidia|o__Bacteroidales|f__Bacteroidaceae|g__Bacteroides|s__caccae</td><td>0.0234</td></tr>
<tr><td>k__Bacteria|p__Firmicutes|c__Clostridia|o__Coriobacteriales|f__Coriobacteriaceae|g__Collinsella|s__aerofaciens</td><td>0.0234</td></tr>
<tr><td>k__Bacteria|p__Firmicutes|c__Clostridia|o__Clostridiales|f__Veillonellaceae|g__Megamonas|s__unclassified</td><td>0.0233</td></tr>
<tr><td>k__Bacteria|p__Proteobacteria|c__Alphaproteobacteria|o__Rickettsiales|f__mitochondria|g__unclassified|s__unclassified</td><td>0.0233</td></tr>
</table>

Supplementary Fig. 6: The individual names and MAEs of bacteria which have (a) top 10 largest MAE and (b) bottom 10 smallest MAE identified in Supplementary Fig. 5.

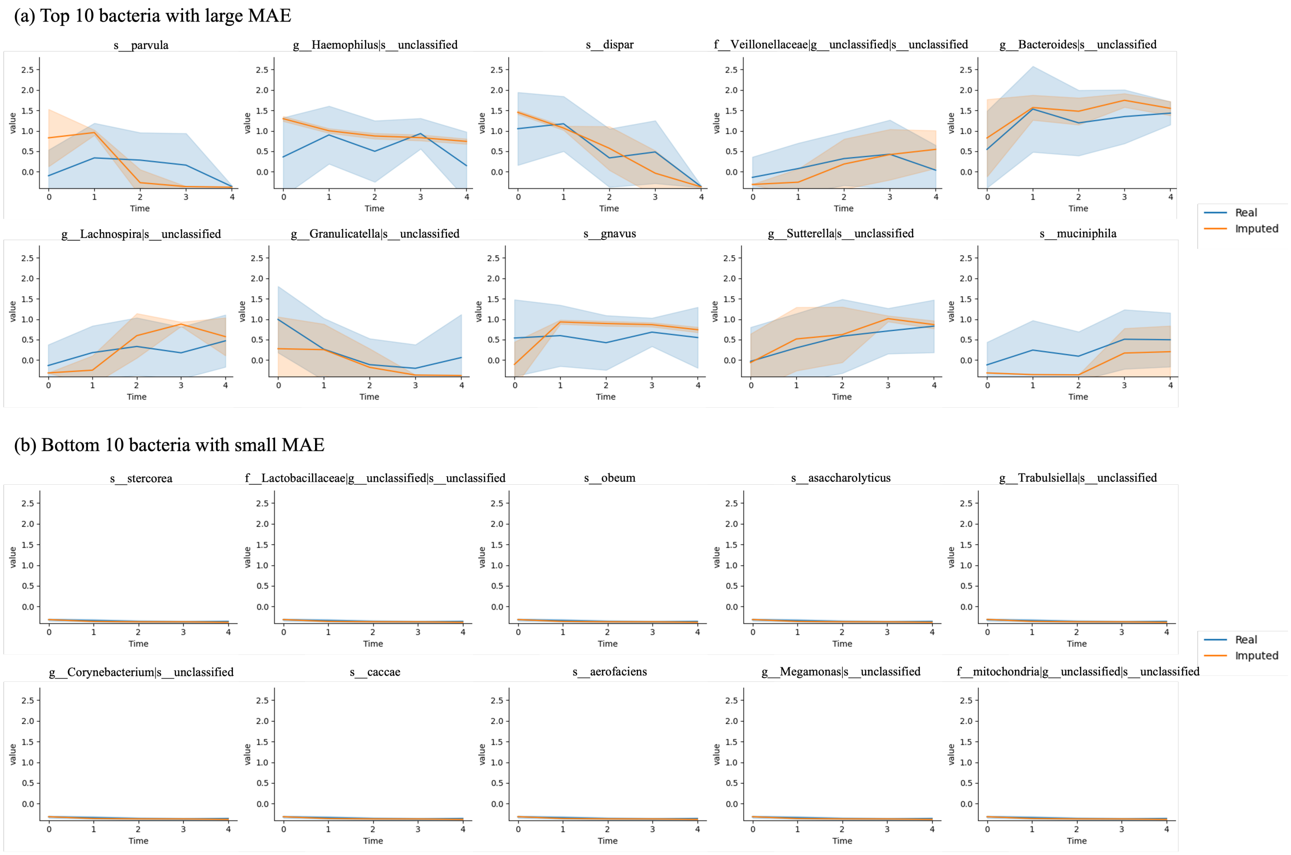

Supplementary Fig. 7: Time-course plot for each bacteria which shows (a) top 10 largest MAE and (b) bottom 10 smallest MAE identified in Supplementary Fig. 5. The line presents the mean abundance (clr-transformed) and the ribbon presents standard deviation.

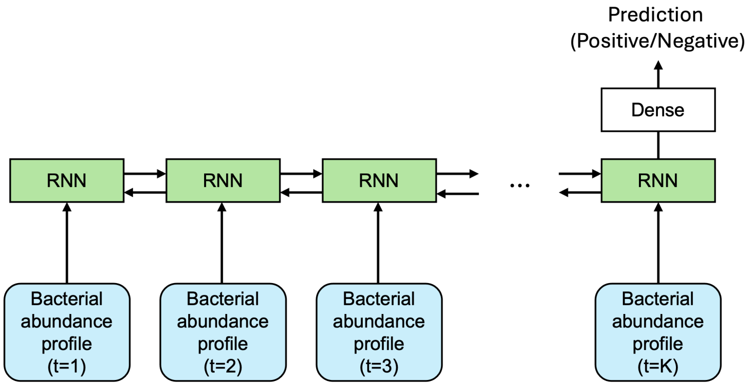

Supplementary Fig. 8: Architecture of the bidirectional RNN-based model used to predict disease presence.

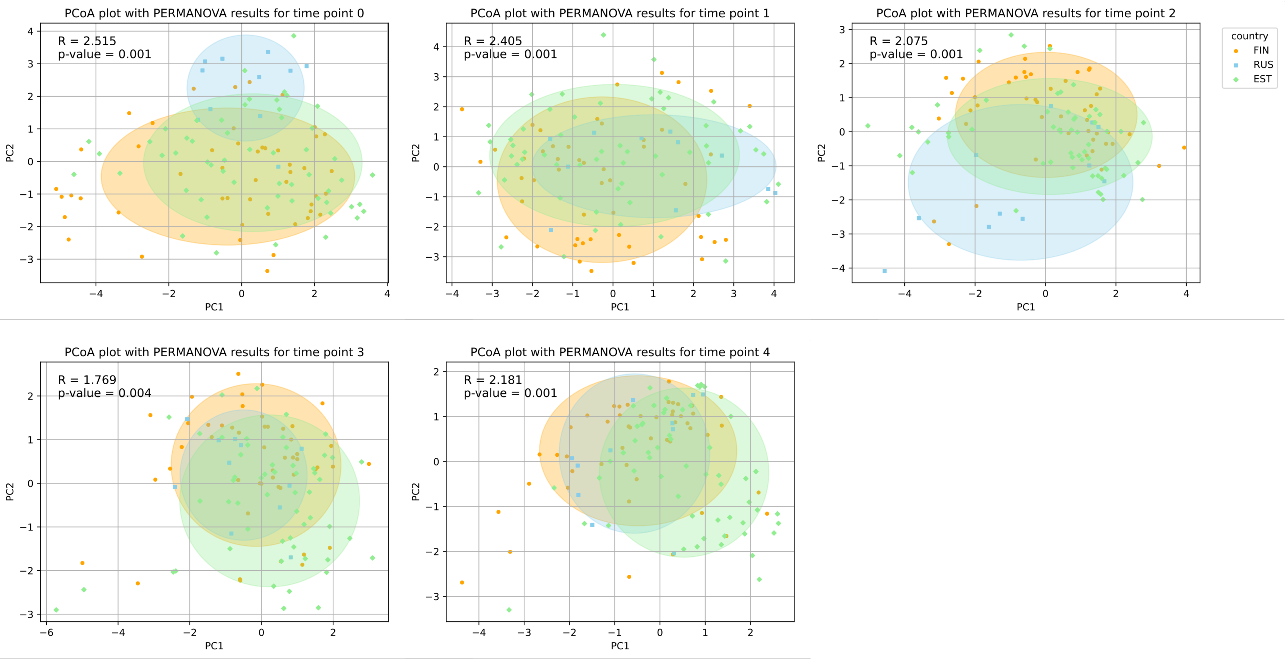

Supplementary Fig. 9: PCoA plots with PERMANOVA results on 16S rRNA profile from the DIABIMMUNE study for country.

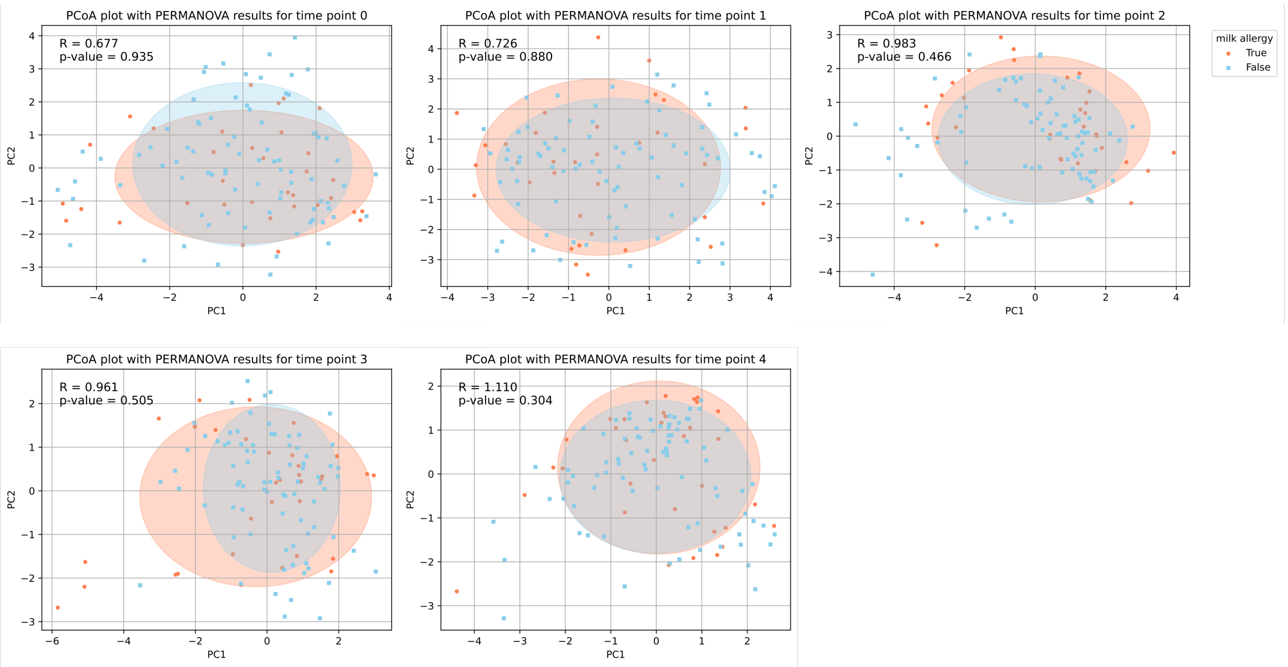

Supplementary Fig. 10: PCoA plots with PERMANOVA results on 16S rRNA profile from the DIABIMMUNE study for presence of milk allergy.

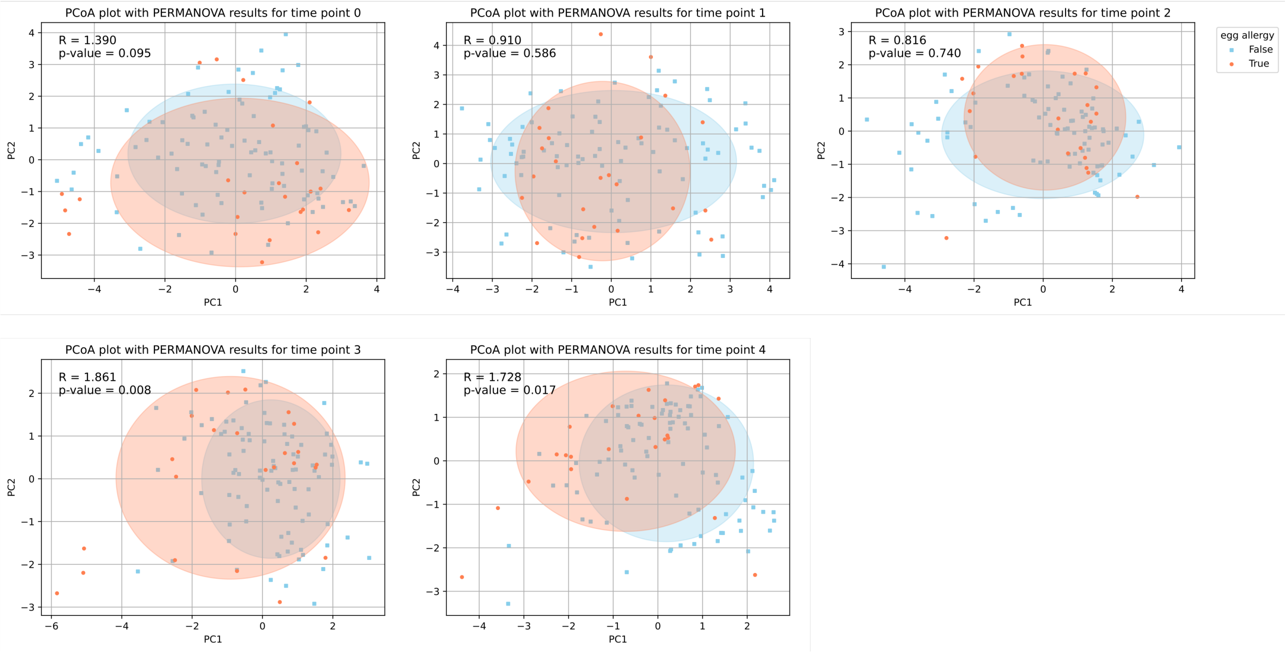

Supplementary Fig. 11: PCoA plots with PERMANOVA results on 16S rRNA profile from the DIABIMMUNE study for presence of egg allergy.

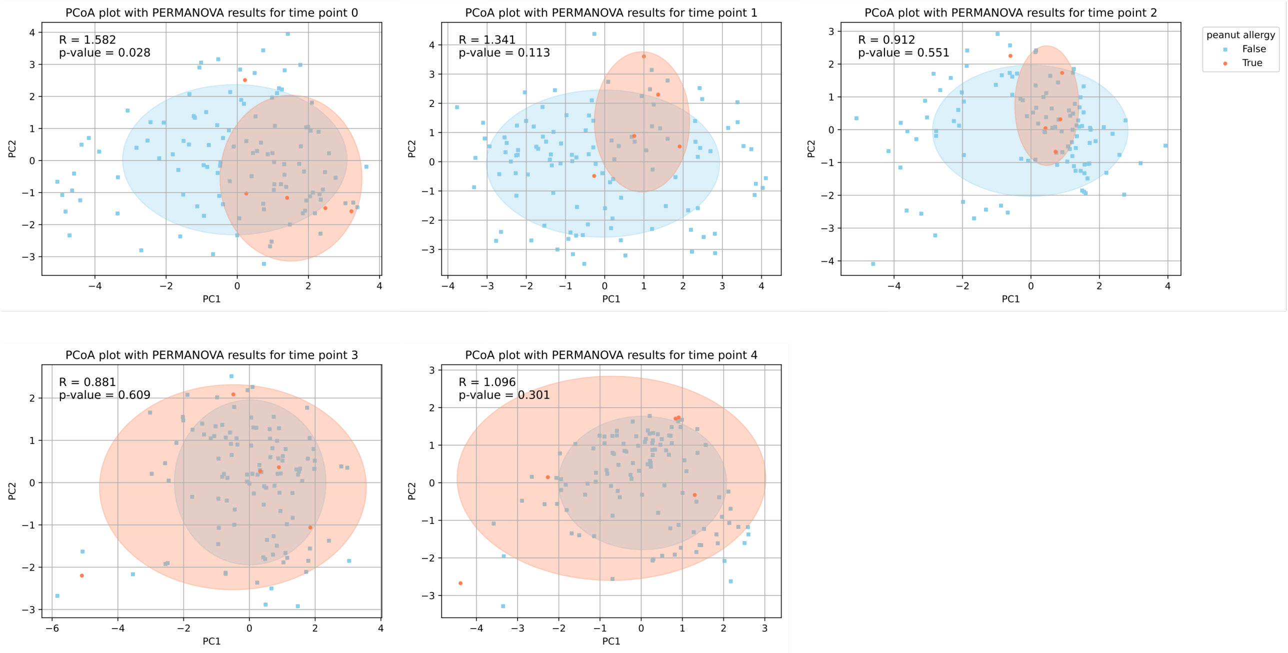

Supplementary Fig. 12: PCoA plots with PERMANOVA results on 16S rRNA profile from the DIABIMMUNE study for presence of peanut allergy.

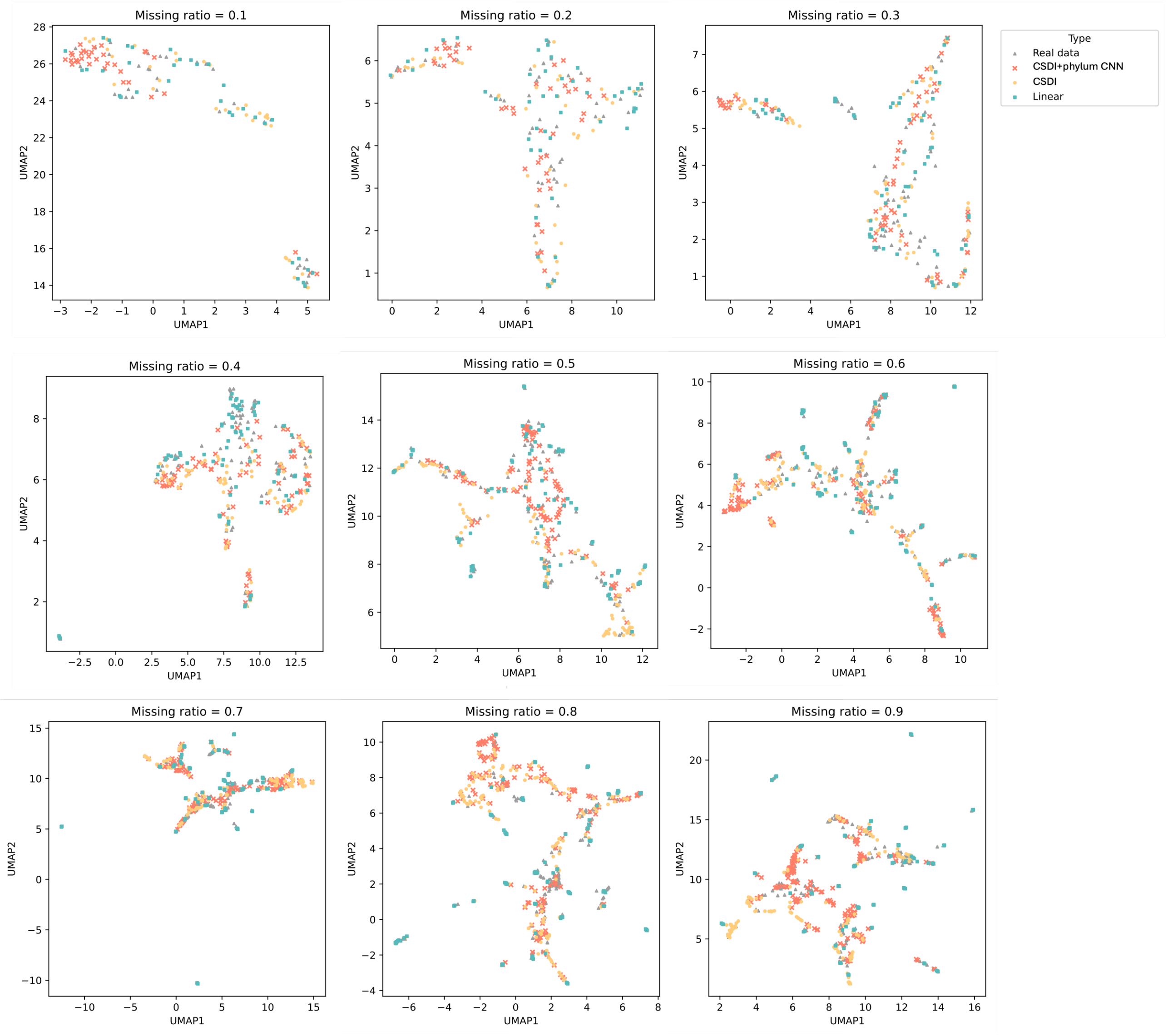

Supplementary Fig. 13: UMAP plots of imputed profiles for the first fold out of 5-fold cross-validation for WGS data from BONUS study.

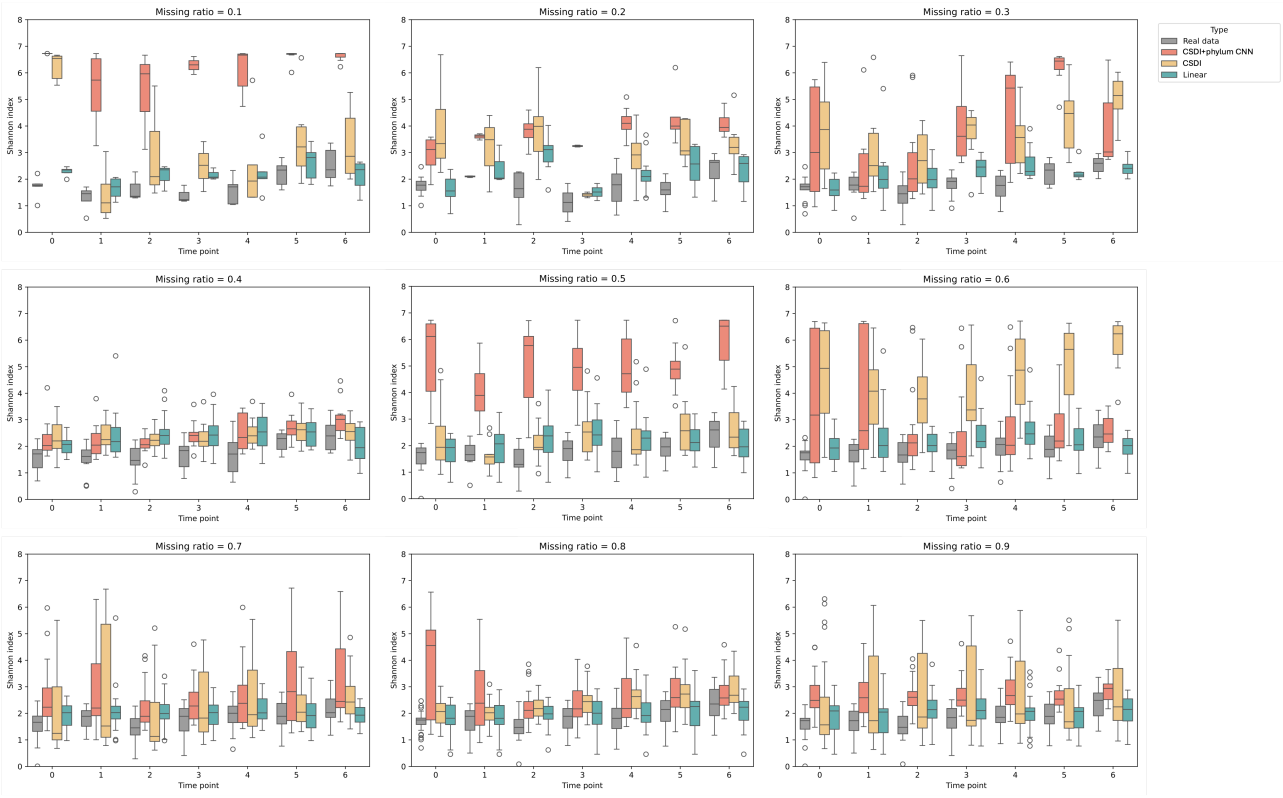

Supplementary Fig. 14: Distribution of **alpha diversity** by time point across various missing ratios for WGS data from BONUS study.

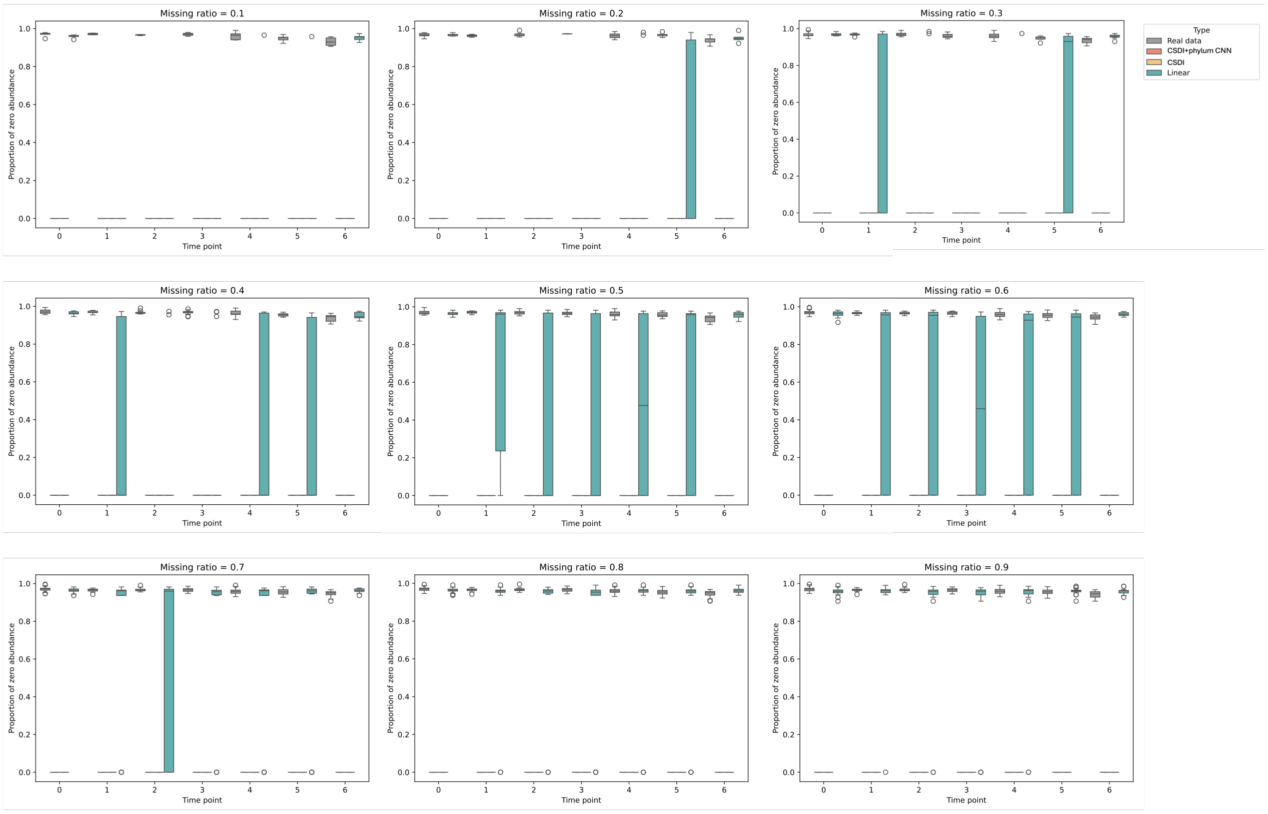

Supplementary Fig. 15: Distribution of **proportion of zero abundance** by time point across various missing ratios for WGS data from BONUS study.

References

[1] Tommi Vatanen, Aleksandar D Kostic, Eva d'Hennezel, Heli Siljander, Eric A Franzosa, Moran Yassour, Raivo Kolde, Hera Vlamakis, Timothy D Arthur, Anu-Maaria Hämäläinen, et al. Variation in microbiome lps immunogenicity contributes to autoimmunity in humans. *Cell*, 165(4):842–853, 2016.

[2] Hillary S Hayden, Alexander Eng, Christopher E Pope, Mitchell J Brittnacher, Anh T Vo, Eli J Weiss, Kyle R Hager, Bryan D Martin, Daniel H Leung, Sonya L Heltshe, et al. Fecal dysbiosis in infants with cystic fibrosis is associated with early linear growth failure. *Nature medicine*, 26(2):215–221, 2020.

[3] Misato Seki, Yao-Zhong Zhang, and Seiya Imoto. Imputing time-series microbiome abundance profiles with diffusion model. In *2023 IEEE International Conference on Bioinformatics and Biomedicine (BIBM)*, pages 914–919. IEEE, 2023.
